# Gut Microbiome Communities Vary Across Translocated Populations of the Seychelles Warbler

**DOI:** 10.1002/ece3.73750

**Published:** 2026-05-29

**Authors:** Sarah F. Worsley, Zoe Crighton, Chuen Zhang Lee, Terry Burke, Jan Komdeur, Hannah L. Dugdale, David S. Richardson

**Affiliations:** ^1^ School of Biological Sciences University of East Anglia Norfolk UK; ^2^ Ecology and Evolutionary Biology, School of Biosciences University of Sheffield Sheffield UK; ^3^ Groningen Institute for Evolutionary Life Sciences (GELIFES) University of Groningen Groningen the Netherlands; ^4^ Faculty of Biological Sciences, School of Biology University of Leeds Leeds UK; ^5^ Nature Seychelles Mahé Republic of Seychelles

**Keywords:** *Acrocephalus sechellensis*, conservation, founder effects, gut microbiome, re‐introduction, translocation

## Abstract

Conservation translocations are an increasingly common tool used to help combat species extinction and global biodiversity loss. However, their success is dependent on a wide range of abiotic and biotic factors. To date, the potential role of host‐associated microbiomes in translocation success has been overlooked despite their fundamental contribution to host health and fitness. Here, we use faecal samples to evaluate how gut microbiome communities vary across the last remnant (source) population, and all four translocated populations (established between 1988 and 2011), of the Seychelles warbler ( *Acrocephalus sechellensis* ). Gut microbiome alpha diversity was lower in all translocated populations compared to the source population on Cousin Island. Gut microbiome composition also varied, with several short‐chain fatty acid producing bacterial families being lost from the core microbiome in some translocated populations; such taxa have been shown to play an important role in maintaining host metabolic health. Furthermore, the two translocated populations that were established the longest time ago, and with the fewest individuals, had reduced inter‐individual gut microbiome variability compared to the source population. While it was not possible to directly assess the specific drivers of these differences due to samples being collected after the translocation event, it is likely that the size of the founding population, subsequent loss of host genetic variation and environmental factors all contribute to shaping gut microbiome variation amongst these populations. Future work should assess whether taxonomic variation translates into differences in gut microbiome function and the possible consequences for host population health and long‐term resilience to environmental change.

## Graphical abstract

Conservation translocations are an increasingly common tool used to help combat species extinction but their success is dependent on a wide range of abiotic and biotic factors. To date, the potential role of host‐associated microbiomes in translocation success has been overlooked despite their fundamental contribution to host health and fitness. Here, we evaluate how gut microbiome communities vary across the last remnant (source) population and all four translocated populations of the Seychelles warbler ( *Acrocephalus sechellensis* ) to better understand the long‐term impacts of translocations on host–microbe interactions.

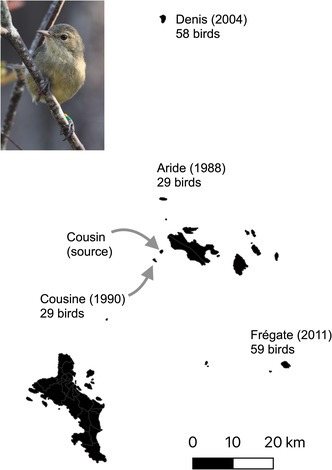

## 1 Introduction

Conservation translocations, involving the deliberate movement of organisms to restore threatened or extirpated wildlife populations (IUCN/SSC 2013), are becoming an increasingly common tool to combat global biodiversity loss (Seddon et al. 2014). However, their success can be highly variable and is dependent on a wide range of abiotic and biotic factors (Berger‐Tal et al. 2020; Bubac et al. 2019). For example, this can include habitat quality at the release site as well as the erosion of genetic diversity and genetic drift occurring in small populations influenced by founder effects (Berger‐Tal et al. 2020; Bubac et al. 2019). However, whilst some factors, such as direct founder effects, have been well‐studied, other potential drivers of inconsistent translocation success have been overlooked. This includes the possible role of host‐associated microbiomes.

The vertebrate gut microbiome is a diverse microbial community that makes fundamental contributions to host biological processes including nutrient acquisition, immunity, development and behaviour (Davidson et al. 2020; Nicholson et al. 2012; Sommer and Bäckhed 2013). In wild animals, gut microbiome composition varies extensively amongst and within populations of the same species (Björk et al. 2022; Grieneisen et al. 2019; Worsley, Davies, et al. 2024). This variation is partially shaped by ecological factors such as habitat type and diet (Baniel et al. 2021; Fackelmann et al. 2021; Worsley et al. 2025). However, it can also be influenced by host traits such as age (Reese et al. 2021), relatedness (Baniel et al. 2022) and host genotype (Davies et al. 2022; Worsley et al. 2022). Studies on natural populations have suggested that such variation can have significant consequences for host health and fitness. For example, inter‐individual gut microbiome differences have been associated with differences in host disease status (Navine et al. 2022), survival (Davidson et al. 2021; Worsley et al. 2021, 2022), and reproductive performance (Leclaire et al. 2022). As such, changes in the gut microbiome resulting from translocation could have significant implications for conservation outcomes. Despite this, studies characterising the gut microbiome of translocated species are lacking and are mainly focussed on animals moved from captivity into wild habitats (Chong et al. 2019; Uren Webster et al. 2020; Wang et al. 2019; Yao et al. 2019, but see Blyton et al. 2023).

Translocations could have negative consequences for the host if mismatches arise between the gut microbiome and the host's new environment. For example, existing gut microbes may lack the enzymes necessary to degrade new dietary components or environmental toxins present at the release site (Blyton et al. 2019; Carthey et al. 2020). Habitat differences and increased host stress could further perturb gut microbiome composition and result in the loss of functionally important microbes (Fackelmann et al. 2021; Stothart et al. 2019). Loss of microbes could also be driven by the population bottleneck associated with translocating a small number of founding individuals. Firstly, founder effects could restrict the pool of host‐associated microbes available for transmission, both in the initial translocated population and across future generations (i.e., a direct bottleneck of the microbiome) (Ørsted et al. 2022). Secondly, since microbiome composition is also partially shaped by host genotype (Davies et al. 2022; Worsley et al. 2022), loss of host genetic variation and increased inbreeding in small translocated populations (i.e., host founder effects) could further disrupt the gut microbiome over future generations (Ørsted et al. 2022). This may be exacerbated if population growth rate is slow and only a subset of individuals contributes to reproduction post‐translocation. Such changes could be irreversible as some microbes don't exist independently of their hosts and, thus, cannot be re‐acquired from the environment (Carthey et al. 2020). Instead, lost microbes may be replaced by less functionally relevant species or pathogenic strains (Carthey et al. 2020).

Importantly the loss of gut microbiome variation (described above) may be exacerbated by the conditions experienced by individuals undergoing translocation. In many translocations, individuals may be quarantined in captivity for extended periods of time and given artificial diets and medication (Kock et al. 2010), all of which have been shown to radically alter the gut microbiome, often with deleterious effects (Dallas and Warne 2023; Ramirez et al. 2020; San Juan et al. 2021). Thus, it is highly probable that these practices may have negative consequences for the microbiome health of translocated individuals and the subsequent population.

Conversely, it is also plausible that gut microbiome plasticity could facilitate host acclimatisation to novel environments and, thus, improve translocation success. Studies on naturally dispersing species, such as yellow baboons ( *Papio cynocephalus* ), have shown that individuals gradually acquire microbes from their new local environment (Grieneisen et al. 2017). Similarly, in humans, dietary change rapidly alters gut microbiome communities (David et al. 2014). Although some microbes may be functionally redundant, the accumulation of new, functionally distinct strains could enable acclimatisation to novel ecological conditions, providing the host with a fitness advantage (Alberdi et al. 2016; Carthey et al. 2020). Such effects have not been well‐studied in natural populations, but faecal transplant experiments in mammals suggest that acquiring novel microbes can alter traits such as host dietary range (Blyton et al. 2019) and toxin degradation capabilities (Kohl et al. 2014). Comparing source and translocated populations provides an opportunity to study the taxonomic and functional plasticity of the gut microbiome in a natural setting.

**FIGURE 1:**
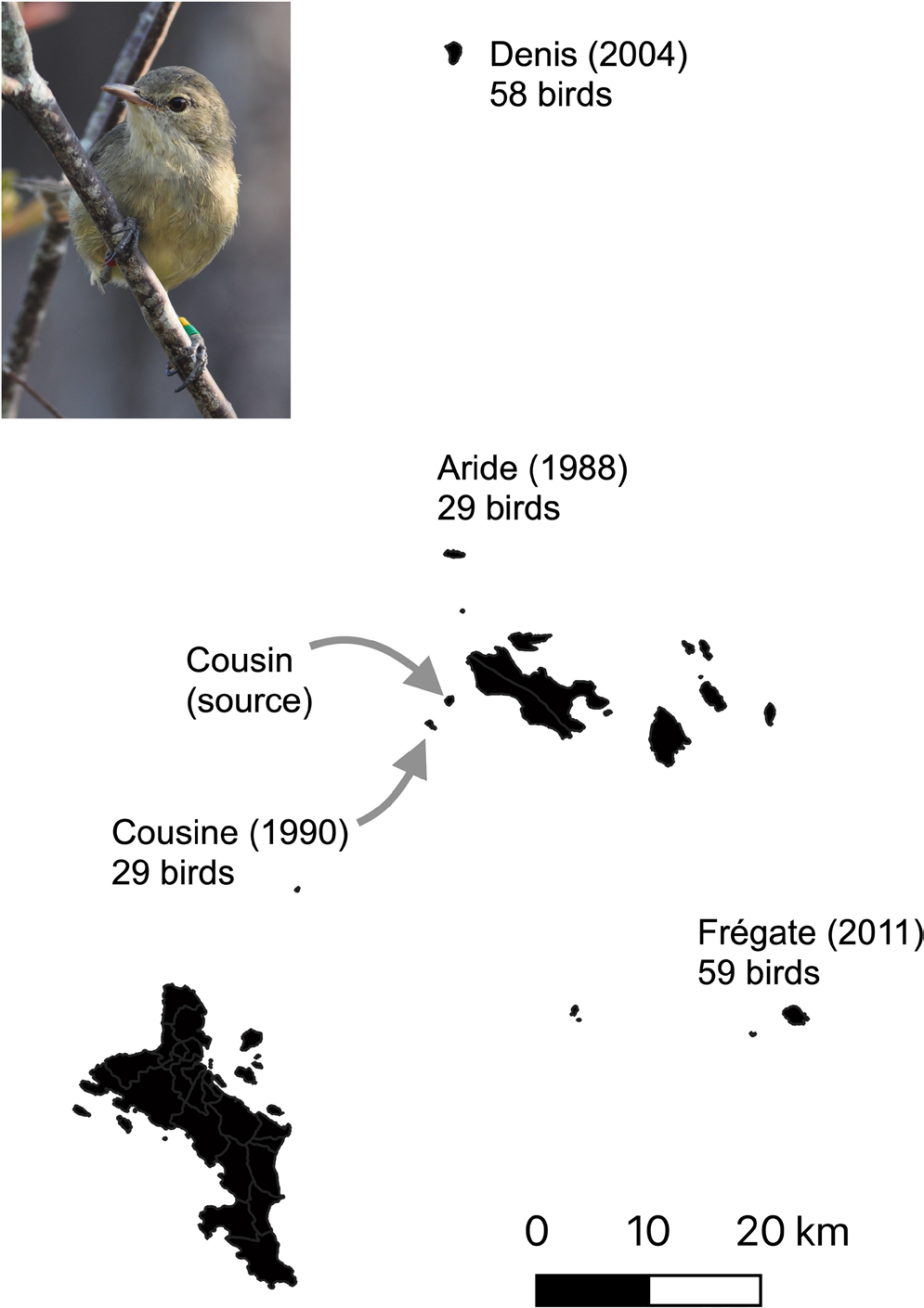
Source and translocated populations of the Seychelles warbler (pictured inset). The year of translocation from the source population on Cousin Island is shown in brackets. Numbers represent the number of founding individuals introduced to each translocated population. Photo credit: Charli S. Davies.

Here, we use faecal samples (collected 2019–2023) from five discrete populations of Seychelles warblers ( *Acrocephalus sechellensis* ) to evaluate whether conservation translocations have long‐term impacts on the bacterial gut microbiome. In the 1960s the Seychelles warbler was close to extinction, with less than 29 individuals remaining on Cousin Island (Penny 1967; Spurgin et al. 2014). Since then, habitat restoration has dramatically increased the size of this population to carrying capacity and it has become the subject of a long‐term ecological and evolutionary study (Davies et al. 2021; Hammers et al. 2015; Richardson et al. 2002; Sparks et al. 2021). As part of this species' conservation plan, individuals have subsequently been successfully translocated from Cousin to four other islands in the Seychelles archipelago (Aride, Cousine, Denis and Frégate, Figure 1) (Komdeur 1994; Richardson et al. 2006; Wright et al. 2014). Subsequent work has shown that while most genetic variation was successfully translocated in each attempt, founder effects have resulted in some loss and structuring of neutral and functional diversity, especially in those populations established with the fewest founders (Wright et al. 2014).

We first test whether gut microbiome alpha diversity (the number of bacterial taxa) differs between the source population on Cousin and translocated populations. Increased alpha diversity can be indicative of a healthier microbiome (Sommer and Bäckhed 2013). We expect alpha diversity to vary amongst populations due to environmental differences across islands. More importantly, we hypothesise that diversity will be lower in translocated populations due to founder effects. This may be because translocating a small number of individuals restricts which microbes are associated with hosts in the new population (i.e., a microbiome bottleneck), or because host genetic erosion (due to founder effects) indirectly reduces microbiome diversity. We further predict that these differences will translate into gut microbiome compositional differences (beta diversity) across populations. Finally, we specifically test which members of the core microbiome on the original source population have been retained or lost in translocated populations; the loss of these could have implications for gut function and host health. Conversely, we also investigate which microbes have been acquired in translocated populations that are absent from the core microbiome on Cousin. These microbes could be beneficial, functionally replacing those microbes that have been lost during translocation, be neutral environmental bacteria, or be deleterious pathogenic taxa.

## 2 Methods

### 2.1 Study Species and Sample Collection

The Seychelles warbler ‐an endemic insectivorous passerine‐ was historically widespread across the islands of the Seychelles; however, habitat loss and the introduction of invasive predators brought it close to extinction in the mid‐20th century (Spurgin et al. 2014). By 1960, there were reportedly fewer than 29 individuals remaining on Cousin Island (4.3315° S, 55.6620° E, 0.29 km^2^) (Penny 1967). Since then, habitat restoration on Cousin has allowed the size of this population to reach a carrying capacity of *ca* 320 adult individuals from 1982 onwards (Hammers et al. 2019; Komdeur and Pels 2005). As part of this species' conservation plan, individuals have subsequently been translocated from Cousin to four other islands in the Seychelles archipelago (Figure 1) (Komdeur 1994; Richardson et al. 2006; Wright et al. 2014). In 1988 and 1990 respectively, 29 birds were translocated to Aride (4°120 S, 55°400 E, 0.68 km^2^) and Cousine (4°210 S, 55°390 E, 0.25 km^2^) (Komdeur 1994). Following this, 58 birds were translocated to Denis (3°480 S, 55°400 E, 1.42 km^2^) in 2004 (Richardson et al. 2006) and 59 birds to Frégate (4°350 S, 55°560 E, 2.19 km^2^) in 2011 (Wright et al. 2014). Birds were translocated without knowledge of the individuals' host genetic variation or gut microbiome variation, but were of approximately equal sex ratios, age structure and body condition and were released in areas of good quality habitat (Komdeur 1994; Richardson et al. 2006; Wright et al. 2014). In each case, birds were not kept captive for longer than 24 h and received no artificial food or medication during translocation (Komdeur 1994; Richardson et al. 2006; Wright et al. 2014). Subsequent monitoring showed that all birds survived the translocations, and that the populations expanded, rapidly reaching carrying capacity on Aride and Cousine, and still growing on both Denis and Frégate (Brown et al. 2023). Current population size estimates are *ca* 1850, 210, 875 and 561 individuals on Aride, Cousine, Denis and Frégate, respectively (Brown et al. 2023). Movement between the populations is absent, except for extremely rare dispersal events between the two closest islands of Cousin and Cousine (*n* = 2 over a 20 years period, Komdeur et al. 2004).

Samples (*n* = 20 in each case) were collected from adult birds during the minor breeding season (January–March) of 2019 on Cousine, the major breeding season (June–September) of 2022 on Denis and Frégate, and the major breeding season of 2023 on Αride, respectively. Samples were derived from equal numbers of males and females and were collected randomly from territories distributed across each island. Samples collected on Cousin Island from equivalent seasons (the minor season of 2019 (*n* = 20) and major season of 2022 (*n* = 31)) were also used.

To collect faecal samples, birds were caught in mist nets and placed into a disposable, flat‐bottomed paper bag containing a sterilised weigh boat protected by a metal grate. This established protocol (Knutie and Gotanda 2018; Worsley, Davies, et al. 2024) allows faecal matter to be collected from the tray whilst reducing contact with the bird's surface. Birds were removed from the bag after defecation or after 30 min. Faecal samples were collected using a sterile flocked swab and placed into a microcentrifuge tube containing 1 mL of absolute ethanol. Control swabs from fieldworker hands and collection bags were also collected at the time of sampling. All samples were stored at 4°C for the remainder of the season before being transferred to −80°C for long‐term storage. Prior to release, a blood sample was also taken from the bird via brachial venipuncture and stored in absolute ethanol at 4°C. DNA was extracted from blood samples and used for molecular sexing via a PCR‐based method (Griffiths et al. 1998; Sparks et al. 2021).

### 2.2 Microbiome Extraction and Sequencing

Genomic DNA was extracted from all faecal and collection control samples using the DNeasy PowerSoil kit (Qiagen) according to a modified version of the manufacturer's instructions (see Davies et al. 2022). Extracted DNA was submitted for 16S rRNA gene amplicon sequencing at the NEOF Centre for Genomic Research (Liverpool, UK). Amplicon sequencing libraries were generated using the V4 primers 515F and 806R (see Davies et al. 2022). Libraries underwent 2 × 250 bp, paired‐end sequencing on an Illumina MiSeq platform. Negative extraction blanks and a ZymoBIOMICS microbial mock community standard (D6300) were also sequenced to identify contaminants, check for batch effects, and assess sequencing success (as described in Worsley, Davies, et al. 2024).

### 2.3 Bioinformatic Processing of Sequencing Data

Sequencing reads were processed using QIIME2 2019.10 (Bolyen et al. 2019). Forward and reverse reads were truncated at 240 bp and low quality base calls were trimmed from the 5′ end using the DADA2 plugin (Callahan et al. 2016). Amplicon sequencing variants (ASVs) were inferred for each sample, followed by dereplication and pair‐end joining. Putative chimeras and singleton reads were also removed. ASVs were then taxonomically classified by training a naïve‐Bayes classifier on the SILVA 132 reference database for 16S rRNA gene sequences. ASVs classified as chloroplast or mitochondria were removed. A mid‐point rooted phylogeny was constructed using MAFFT (Katoh 2002) and the Fast Tree (Price et al. 2009) approach. The final ASV, taxonomy, and tree files were exported from QIIME2 into R 4.2.2 (R Core Team 2020).

Files were further processed using *phyloseq* 1.42.0 (McMurdie and Holmes 2013). ASVs were filtered to remove non‐bacterial sequences and those unassigned at phylum level. Potential contaminants were also identified and removed from faecal samples using the prevalence method in *decontam* 1.18.0 (Davis et al. 2018). This method identifies putative contaminants by testing for increased prevalence across negative extraction blanks and collection controls compared to true samples. As a final filtering step, ASVs with fewer than 50 reads across all samples were removed prior to downstream analysis (accounting for ~1% of all reads) as these may represent possible sequencing errors. A cut‐off of 50 reads was chosen based on the presence of low abundance ASVs (< 50 reads) in positive controls. After filtering, 8141 ASVs were detected across 131 faecal samples (mean ASVs per sample = 220.25 ± 10.56 SE). These ASVs clustered into 169 bacterial families (Figure S1).

### 2.4 Statistical Analyses

#### 2.4.1 Alpha Diversity

Samples were rarefied to a depth of 8000 reads prior to calculating alpha diversity metrics based on rarefaction curves which indicated a sample completeness of > 95% at this depth. Shannon diversity and observed ASV richness were calculated using *phyloseq* 1.42.0 (McMurdie and Holmes 2013). Faith's phylogenetic diversity (PD) was calculated using *picante* 1.8.2 (Kembel et al. 2010). Linear models with a gaussian distribution were constructed using *stats* 4.2.2 to test whether Shannon diversity and Faith's PD differed between translocated and source populations. A generalised linear model with a negative binomial distribution was used to model observed ASV richness (using *MASS* 7.3.58.3) which was over‐dispersed. In these models, all samples from translocated populations were grouped into one category (‘translocated’) and all samples from Cousin were merged into one ‘source’ category. Island type (translocated or source) was included as an independent variable in the analysis, as well as sex (male or female), time of day (minutes since sunrise), and time stored at 4°C in the field (all previously shown to influence the warbler gut microbiome (Worsley, Davies, et al. 2024)). In this model, island type was partially confounded by season since all translocated populations, apart from Cousine, were sampled in the major season whereas Cousin was sampled in both major and minor seasons. Thus, we also repeated these models, but with an individual ‘population’ term instead of ‘island type’. The ‘population’ term included samples collected on Cousine, Aride, Denis, Frégate and Cousin (2019 and 2022 separately) as individual factor levels. This allowed us to determine whether sample alpha diversity differed between the major and minor seasons on Cousin (indicating a season effect) and between translocated populations and samples from Cousin collected in the equivalent season (an island effect). In this second set of models, the overall influence of population on alpha diversity was tested via car::Anova type II tests on the fitted model. Post hoc Tukey tests were then used to check for differences amongst specific population pairs.

#### 2.4.2 Beta Diversity (Composition)

Unrarefied reads were used in beta diversity analyses. Rare taxa occurring in ≤ 2 individuals were removed prior to analysis. Sample reads were then transformed using the centred log ratio (CLR) transformation in *microbiome* 1.20.0; this transformation controls for the compositional nature of microbiome data (Gloor et al. 2017). To quantify whether overall gut microbiome composition varied across populations, a marginal permutational analysis of variance (PERMANOVA, 9999 permutations) was performed using the *adonis2()* function within *vegan* 2.6.6.1 (Okansen et al. 2020). This used a matrix of pairwise sample Aitchison distances calculated using the CLR transformed ASV abundances as input. Population (Cousine, Aride, Denis, Frégate, Cousin 2019 or Cousin 2022), sex, time of day and time stored at 4°C were included as independent variables. Post hoc pairwise PERMANOVAs were performed using *pairwiseAdonis* 0.4.1 (Martinez Arbizu 2017). A betadisper test was performed using *vegan* 2.6.6.1 to assess whether the level of inter‐individual gut microbiome variation differed amongst populations (Anderson 2001; Okansen et al. 2020).

#### 2.4.3 Differences in the Core Bacterial Microbiome Across Populations

The core gut microbiome was calculated at the level of bacterial family for each population separately using the *core()* function in *microbiome* 1.20.0. Core microbes were defined as bacterial families that had a total abundance of > 0.1% across samples and were present in > 50% of individuals within a population (Davies et al. 2022; Risely 2020). We first quantified which core families in the Cousin population (2019 and 2022 samples combined) were absent from the core microbiome on each of the other islands (i.e., present in < 50% individuals in each translocated population). Second, we assessed which bacterial families formed part of the core microbiome in translocated populations but were absent from the Cousin core microbiome; these taxa may functionally replace those that have been lost following translocation, or represent new environmentally‐derived and/or pathogenic taxa.

#### 2.4.4 Differential Abundance Tests and Indicator Analysis

An Analysis of Compositions of Microbiomes with Bias Correction (ANCOM‐BC) (Lin and Peddada 2020) was used to identify bacterial families that significantly differed in abundance amongst populations (Cousine, Aride, Denis, Frégate, Cousin 2019 or Cousin 2022) whilst controlling for the effects of host sex, time of day and sample storage time at 4°C. The ‘fdr’ method was used to correct *p*‐values for multiple testing and a cut‐off of *p*_*adj*_ < 0.05 was used to define taxon significance. ANCOM‐BC also identifies structural zeros whereby a taxon is considered differentially abundant because it is completely absent in at least one population. These taxa are not included in statistical significance tests as they give spurious log fold change estimates but can be used to identify patterns of presence/absence across populations (Lin and Peddada 2020). Thus, both statistically significant bacterial families and structural zeros are reported to better understand compositional differences across islands.

Finally, an indicator analysis was conducted using *labdsv* 2.1.0 to determine the specificity and fidelity of bacterial ASVs to each population (Dufrêne and Legendre 1997; Roberts 2023). In this analysis, an indicator score is calculated for each ASV/population combination. Scores are based on the prevalence and average relative abundance of each ASV within each population. For each ASV, the highest indicator score is retained, and their statistical significance is assessed via permutation whereby ASVs are randomised across groups generating a null distribution (Dufrêne and Legendre 1997; Roberts 2023). An indicator score of one would indicate that an ASV is equally abundant in all samples from one population but effectively absent in other populations, whereas a score of zero would suggest approximately even abundances across samples from all populations. ASVs with indicator scores of > 0.4 and with *p*‐values of < 0.05 were considered indicative of populations.

## 3 Results

### 3.1 Gut Microbiome Alpha Diversity

**FIGURE 2:**
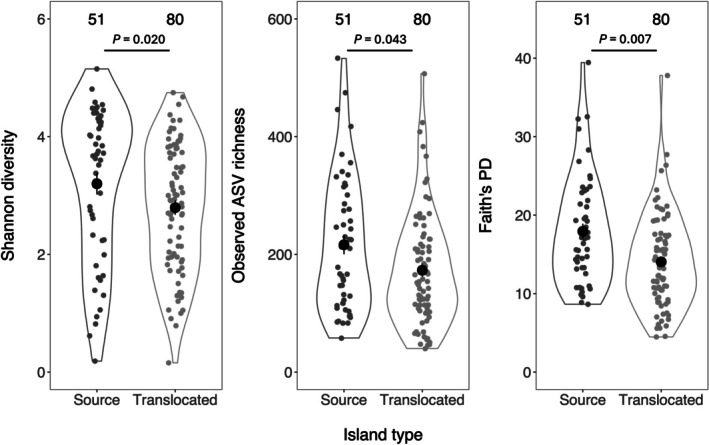
Gut microbiome alpha diversity in the source and translocated populations of the Seychelles warbler. Grey points represent individual gut microbiome samples (*n* = 51 in the source population; *n* = 80 from the four translocated populations). Black points represent the mean ± SE. *p*‐values are derived from linear models (see Table S1 for full results).

Alpha diversity was significantly lower in translocated populations compared to the source population for all diversity metrics when samples were clustered into two categories (source versus translocated samples: *p* < 0.05, Figure 2, Table S1). There was also a significant difference in alpha diversity amongst populations when each population was included as a separate identity (*p* < 0.05, Table S2). Post hoc comparisons of separate translocated islands and seasons on Cousin Island were generally consistent with diversity being lower in translocated populations although this varied across alpha diversity metric and populations (Figure S2). The Faith's PD of samples from Frégate was significantly lower than samples collected during the minor and major seasons on Cousin Island (*p*_*adj*_ < 0.05, Figure S2, Table S3) and samples from Cousine had significantly lower Shannon diversity compared to those from the 2022 Cousin major season (*p*_*adj*_ < 0.05, Figure S2, Table S3). Diversity also tended to be lower on Denis and Aride compared to the equivalent major season on Cousin regardless of the diversity metric used (Figure S2) however, these pairwise comparisons were not significant (Table S3), likely due to the small sample sizes for each island and the large number of post hoc comparisons conducted. Host sex, time of day and sample storage time at 4°C were not significantly associated with gut microbiome alpha diversity in any model (Tables S1 and S2).

### 3.2 Gut Microbiome Beta Diversity (Composition)

**FIGURE 3:**
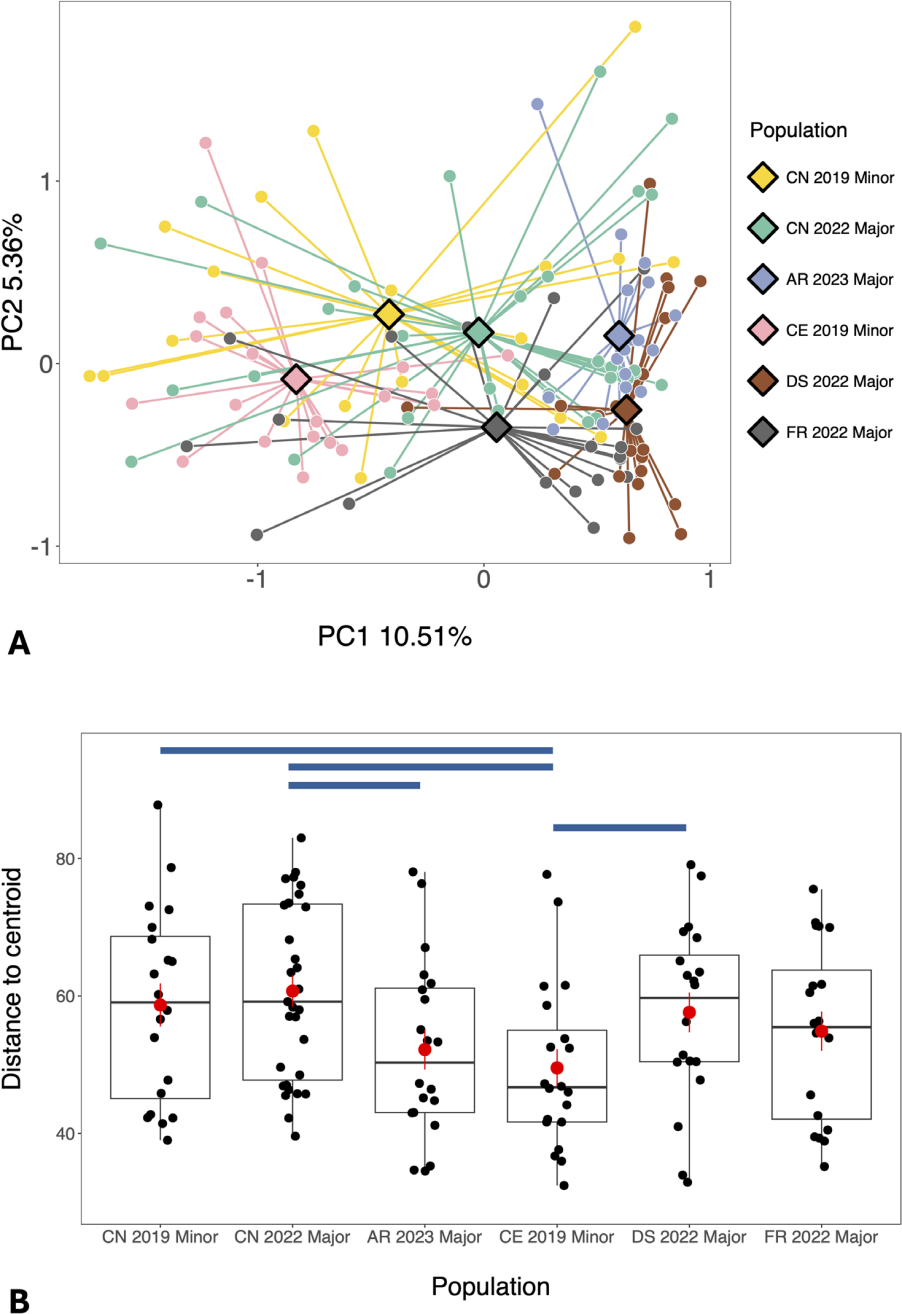
Differences in bacterial gut microbiome composition across populations of the Seychelles warbler. These were visualised using (A) a Principal Components Analysis (PCA) of Euclidean distances calculated using CLR‐transformed ASV abundances. Points represent individual gut microbiome samples and diamonds represent group centroids. Principal components one and two explained 10.51% and 5.36% of the variation in gut microbiome composition, respectively. Differences in (B) gut microbiome compositional variability (distance to centroid) across populations were assessed using a Betadisper test (*p* = 0.039). Red points represent means ± SE. Points represent individual samples, and boxes encompass the interquartile (25%–75%) range. The median is marked by a horizontal line and whiskers extend to 1.5× the interquartile range. Blue lines indicate a significant difference between populations in a post hoc pairwise test (*p*_*adj*_ < 0.05) (see Table S5). In both plots, Cousin (CN) 2019 Minor = 31 samples, all other populations/sampling events had 20 samples. AR, Aride; CE, Cousine; CN, Cousin; DS, Denis; FR, Frégate. The year (2019, 2022 or 2023) and season (Major/Minor) in which samples were collected is also given.

Gut microbiome composition differed significantly across populations in a PERMANOVA analysis (Table 1) with population explaining 11% of the overall variation in composition (*R* ^2^ = 0.11, Table 1). Corresponding with this, all islands (and different sampling events on Cousin Island) formed clusters and had separate group centroids on a PCA plot (Figure 3a). Samples collected from Cousine and Cousin during the minor season of 2019 had lower PC1 axis scores (i.e., they clustered to the left side of the PCA) whereas populations sampled in the major seasons of 2022 and 2023 had higher PC1 scores (Figure 3a). This suggests that there may be some influence of sample year (or season) on gut microbiome composition. However, even within years, samples from individual populations tended to group together on the PCA (Figure 3a) suggesting that island‐specific factors are likely to be driving differences in gut microbiome composition. Indeed, gut microbiome composition differed significantly (*p*_*adj*_ < 0.05) across all pairs of populations/sampling events in post hoc pairwise PERMANOVA analyses (Table S4). The time of day that samples were collected and sample storage time at 4°C were also significantly associated with gut microbiome composition, explaining 1.1% (*p* = 0.012) and 1.4% (*p* = 0.002) of microbiome variation, respectively (Table 1). In contrast, host sex was not associated with gut microbiome beta diversity (*p* = 0.819, Table 1).

**TABLE 1:** A PERMANOVA analysis of gut microbiome variation across different populations of the Seychelles warbler.

Predictor	df	*R* ^2^	*F*	*p*
**Population**	**5**	**0.110**	**3.161**	**< 0.001**
Sex	1	0.006	0.858	0.819
**Time of day**	**1**	**0.011**	**1.597**	**0.012**
**Storage time**	**1**	**0.014**	**1.982**	**0.002**

*Note:* The analysis was performed using Aitchison distances calculated using centred log ratio (CLR)‐transformed amplicon sequencing variant (ASV) abundances. Significant predictors (*p* < 0.05) are shown in bold. *N* = 20 samples from Cousine, Aride, Denis, Frégate and Cousin 2019, and *N* = 31 samples from Cousin 2022 were included in the analysis.

A Betadisper test showed that the amount of inter‐individual gut microbiome variance found within a population also differed across islands (*p* = 0.039). In particular, pairwise tests showed that populations founded with the smallest number of individuals had less compositional variability than the source population; Cousine had significantly lower inter‐individual compositional variance (*p*_*adj*_ < 0.05, Table S5, Figure 3b) compared to both 2019 and 2022 samples from Cousin, whilst Aride had significantly lower inter‐individual variability (*p*_*adj*_ = 0.026, Table S5, Figure 3b) compared to Cousin samples collected in the equivalent major season in 2022.

### 3.3 Shared and Unique Microbes Across Populations

A total of 25 core (> 0.1% total relative abundance and found in > 50% of samples) bacterial families were identified in the Cousin Island population (Table 2a). In comparison, 19 core families were identified on Cousine, 22 on Aride, 23 on Denis and 19 on Frégate (Table 2a). Of the 25 core families on Cousin, 11 (44%) were absent from the core gut microbiome of birds sampled on Cousine (Table 2a). Similarly, 8 (32%) were absent from the core microbiome on Aride, 7 (28%) on Denis and 9 (36%) on Frégate (Table 2a). Whilst these bacterial families were absent from the core microbiome in translocated populations, they still maintained a non‐zero prevalence (Table 2a), indicating that translocation was associated with a reduced frequency of core microbes from the source population rather than a complete loss of these bacterial taxa.

**TABLE 2:** Core gut microbiome bacterial families identified in source and translocated populations.

(A) Core bacterial families on CN Island						
Phylum	Family	CN core	AR core	CE core	DS core	FR core
Firmicutes	Enterococcaceae	0.96	0.95	0.95	0.95	1
Firmicutes	Streptococcaceae	0.71	0.7	0.95	0.7	0.55
Firmicutes	Ruminococcaceae	0.69	0.25	0.95	0.8	0.65
Firmicutes	Lachnospiraceae	0.78	0.35	1	0.7	0.8
Firmicutes	Christensenellaceae	0.57	0.25	1	0.55	0.55
Verrucomicrobia	Akkermansiaceae	0.57	0.15	0.9	0.4	0.4
Planctomycetes	Gemmataceae	0.61	0.6	0.25	0.35	0.25
Chloroflexi	JG30‐KF‐CM45	0.75	0.8	0.25	0.55	0.3
Actinobacteria	Micrococcaceae	0.71	0.9	0.7	0.8	0.55
Actinobacteria	Microbacteriaceae	0.92	1	0.8	0.85	0.9
Actinobacteria	Kineosporiaceae	0.67	0.8	0.25	0.3	0.5
Actinobacteria	Pseudonocardiaceae	0.78	0.8	0.3	0.8	0.85
Actinobacteria	Mycobacteriaceae	0.61	0.55	0.35	0.4	0.3
Actinobacteria	Nocardiaceae	0.80	0.8	0.45	0.8	0.75
Actinobacteria	Propionibacteriaceae	0.61	0.7	0.45	0.55	0.65
Actinobacteria	Nocardioidaceae	0.55	0.55	0.35	0.4	0.65
Bacteroidetes	Tannerellaceae	0.59	0.1	0.95	0.25	0.4
Proteobacteria	Enterobacteriaceae	0.98	0.95	1	0.95	1
Proteobacteria	Burkholderiaceae	0.51	0.35	0.15	0.55	0.3
Proteobacteria	Xanthomonadaceae	0.59	0.5	0.4	0.45	0.4
Proteobacteria	Rhodospirillaceae	0.67	0.1	1	0.65	0.65
Proteobacteria	Acetobacteraceae	0.65	0.55	0.3	0.8	0.65
Proteobacteria	Beijerinckiaceae	0.86	0.9	0.75	0.85	0.95
Proteobacteria	Rhodobacteraceae	0.78	0.9	0.65	0.55	0.3
Proteobacteria	Rhizobiaceae	0.92	1	1	0.9	0.9

(B) Core bacterial families on other islands but absent from CN core						
Phylum	Family	CN core	AR core	CE core	DS core	FR core
Firmicutes	Family XIII	0.49	0.05	0.7	0.35	0.45
Bacteroidetes	Bacteroidaceae	0.45	0.05	0.9	0.4	0.35
Bacteroidetes	Dysgonomonadaceae	0.45	0	0.75	0.1	0.35
Bacteroidetes	Rikenellaceae	0.35	0	0.7	0.15	0.25
Proteobacteria	Desulfovibrionaceae	0.43	0.1	0.75	0.7	0.55
Firmicutes	Leuconostocaceae	0.18	0.8	0.1	0.5	0.25
Patescibacteria	Uncultured bacterium	0.47	0.55	0.35	0.25	0.45
Patescibacteria	Saccharimonadaceae	0.43	0.8	0.4	0.6	0.4
Planctomycetes	Isosphaeraceae	0.43	0.55	0.1	0.35	0.35
Actinobacteria	Corynebacteriaceae	0.37	0.7	0.05	0.65	0.8
Proteobacteria	Desulfobacteraceae	0.02	0.05	0	0.55	0.15
Firmicutes	Lactobacillaceae	0.29	0.45	0.25	0.55	0.6

*Note:* Colours reflect the presence (blue) or absence (green) of a bacterial family from the core microbiome of a population. (A) The 25 core bacterial families identified on Cousin Island in the core microbiome of other translocated populations. (B) Bacterial families found in the core microbiome of at least one translocated population but absent from the Cousin core. Numbers represent the proportion of samples containing each bacterial family per population (i.e., the prevalence). Core families are those with a total relative abundance > 0.1% and prevalence > 50% (0.5) in a population.

Abbreviations: AR, Aride; CE, Cousine; CN, Cousin; DS, Denis; FR, Frégate.

Most of the bacterial families that were lost from the core microbiome of translocated populations were aerobic members of the phyla Actinobacteria and Proteobacteria (Table 2a). However, on Aride, several anaerobic members of the phylum Firmicutes (*Ruminococcaceae*, *Lachnospiraceae* and *Christensenellaceae*) as well as the family *Akkermansiaceae* were also absent from the core microbiome (Table 2a). Only one family (*Xanthomonadaceae*) was absent from the core of all translocated populations. Conversely, seven families were shared across the core gut microbiome of all populations (Table 2a); these were *Enterococcaceae*, *Streptococcaceae*, *Micrococcaceae*, *Microbacteriaceae*, *Enterobacteriaceae, Beijerinckiaceae* and *Rhizobiaceae*.

We also assessed which bacterial families formed part of the core microbiome of other populations but were not found in the core microbiome on Cousin (Table 2b); these taxa may functionally replace those that have been lost following translocation, or represent new environmentally‐derived or pathogenic taxa. Of the 19 core families on Cousine, 5 were not identified in the core microbiome on Cousin (Table 2b). Similarly, 5 (out of 22) core families on Aride, 5 (out of 23) on Denis and 3 (out of 19) on Frégate were not identified in the Cousin core, respectively (Table 2b).

Considering all bacterial families, an ANCOM‐BC model identified 20 taxa that significantly differed in abundance between populations (*p*_*adj*_ < 0.05, Table S6). *Leuconostocaceae* was more abundant on Aride than on all other islands (Table S6). The families *JG30‐KF‐CM45*, *Kineosporiaceae* and *Rhodobacteraceae* were also more abundant on Aride than on Denis, whereas *Desulfovibrionaceae* was more abundant on Denis than Aride (Table S6). The remaining families (*Akkermansiaceae, Bacteroidaceae, Desulfovibrionaceae, Dysgonomonadaceae, Rhodospirillaceae, Rikenellaceae, Ruminococcaceae* and *Tannerellaceae*) were all more abundant on Cousine than on other translocated islands (Table S6). The majority of these taxa were also identified as being unique to the core microbiomes of these islands (Table 2b).

In addition to significant differences in abundance across populations, 112 families were identified as having structural zeros, meaning they are completely absent in at least one population (Figure S3). The number of absent families was generally higher in translocated populations (Frégate = 84, Aride and Denis both = 58) compared to the source population on Cousin (44 and 31 in 2019 and 2022, respectively), with the exception of Cousine which had a similar number to Cousin (39 absent families). Most of the families that were absent in translocated populations were present in samples from at least one sampling season on Cousin (Frégate = 77, Aride = 50, DS = 53 and CE = 34; Figure S3). These results are consistent with the lower levels of gut microbiome alpha diversity identified in translocated populations (Figure 2 and Figure S2; Tables S1–S3). However, which taxa were lost in translocated families varied across populations, with only two families, *Parachlamydiaceae* and one uncultured family of *Actinobacteria*, being completely absent in all translocated populations but present on Cousin (Figure S3). In contrast to lost taxa, only a small number of families (Frégate = 4, Aride = 3, Cousine and Denis both = 6) were found on translocated islands but were completely absent in Cousin samples. Overall, 44% of all families identified as having structural zeros were aerobic members of the phyla *Actinobacteria* and *Proteobacteria*, suggesting that patterns of presence/absence are predominantly driven by environmentally derived taxa.

Finally, an indicator analysis at the ASV level was largely consistent with comparisons of core families and results of differential abundance tests (Table S7). Indeed, ASVs from families that were present in the core gut microbiome of specific translocated populations but absent from the core microbiome of Cousin Island birds (Table 2b) and were differentially abundant in ANCOM‐BC tests (Table S6) tended to have significant indicator scores (Table S7). For example, three ASVs in the family *Leuconostocaceae* had significant scores (indicator score > 0.4, *p* < 0.05) on Aride suggesting they were indicative of this population (Table S7); *Leuconostocaceae* was also identified as a member of the core microbiome on this island but not in other populations (Table 2b) and was more abundant on Aride in differential abundance tests (Table S6). However, in some instances, unique ASVs from the same bacterial family were indicators in different populations. For example, Cousin, Cousine and Denis each had a different ASV in the family *Lachnospiraceae* that was indicative for that population (Table S6). Similarly, two different ASVs in the *Enterobacteriaceae* were indicators on Cousin and Cousine, respectively (Table S6).

## 4 Discussion

We compared gut microbiome samples from the source and all four translocated populations of the Seychelles warbler to better understand how such translocations may alter host‐microbial interactions. There was a reduction in gut microbiome alpha diversity across translocated populations compared to the source population on Cousin Island, and differences in gut microbiome composition across islands. Additionally, the two translocated populations established with the smallest numbers of founders and the longest time ago (Cousine and Aride) demonstrated reduced levels of compositional variability compared to the other populations. More than 50% of bacterial families identified in the Cousin Island core gut microbiome were identified in the core microbiome of translocated populations. However, various members of the Cousin core were missing from the core microbiome of translocated populations whilst other bacterial families were completely unique to translocated islands.

### 4.1 Gut Microbiome Variation and Founder Effects

The reduction in alpha diversity and change in gut microbiome composition identified across translocated warbler populations could be driven by a number of different factors. Firstly, given that microbiome structure is partially shaped by host genotype (Davies et al. 2022; Grieneisen et al. 2021; Smith et al. 2015; Worsley et al. 2022), it is plausible that this variation could be related to genetic differences that have accumulated amongst populations following translocation. As outlined earlier, low levels of genetic differentiation exist amongst the warbler populations, with some loss of genetic diversity at neutral microsatellite and MHC loci in the translocated populations (Wright et al. 2014). This is most pronounced in the Aride and Cousine populations, established the longest time ago, and with the fewest founders (Wright et al. 2014). Corresponding with this, as well as compositional differences, we identified reduced inter‐individual gut microbiome variability on Aride and Cousine compared to other populations. Furthermore, many of the bacterial families that changed in prevalence and/or abundance on translocated populations have previously been related to variation in MHC genotype across Seychelles warblers (Davies et al. 2022). For example, the abundances of almost all core actinobacterial families, as well as several families in the phylum *Firmicutes*, have previously been related to the presence/absence of MHC alleles in warblers on Cousin Island (Davies et al. 2022). Thus, it is possible that gut microbiome differences identified across translocated populations could have been partially driven by the loss of host genetic diversity resulting from founder effects.

Founder effects may also directly influence the microbiome since a reduction to host population size could restrict the pool of host‐associated microbes available for transmission in the initial translocated population and future generations. Indeed, laboratory experiments on *Drosophila* have shown that population bottlenecks constrain microbiome richness and result in a core microbiome that is a compositional subset of the original source population (Ørsted et al. 2022). Thus, the reduction in bacterial alpha diversity in translocated populations and the reduced compositional variability on Aride and Cousine could be due to the direct effect of the gut microbiota community being bottlenecked rather than a by‐product of host genetic differences due to host founder effects (or a combination of the two).

Translocated warblers were not kept captive for longer than 24 h and received no artificial food or medication during translocation (Komdeur 1994; Richardson et al. 2006; Wright et al. 2014). Thus, it is unlikely that changes to the gut microbiome of translocated birds were caused by the translocation protocol. However, in other translocations, animals are often kept for much longer periods in captivity and may experience conditions that are far removed from those in their source environment (Kock et al. 2010). In such cases, there may be larger impacts on the gut microbiome of translocated individuals with possible implications for their successful establishment and the health of these populations downstream (Dallas and Warne 2023).

### 4.2 Gut Microbiome Variation and Environmental Factors

Gut microbiome differences in translocated birds could also arise due to environmental variation amongst islands. Both Cousin Island and Aride are protected nature reserves inhabited by a high density of seabirds, whilst Frégate, Denis and Cousine, have less seabirds but more humans (including tourist accommodation/hotel facilities) and, on Denis, considerable livestock. Previous studies on wild systems have demonstrated a strong link between environmental factors and microbiome structure (e.g., Baniel et al. 2021; Fackelmann et al. 2021; Smith et al. 2015; Stothart et al. 2019). Such patterns can be driven by differential exposure to, and dietary uptake of, microbes from the external environment, or via host behavioural/stress responses that indirectly alter microbiome composition (Smith et al. 2015; Stothart et al. 2019). It is currently unknown whether warblers have different diets across islands, but it is possible that variation in insect communities alters exposure to dietary microbes. However, although environmental differences are likely to have resulted in some of the observed compositional differences across islands, none of the factors we identify above (the density of seabirds, or presence of humans/livestock) are consistent with the patterns of alpha diversity and compositional variation we see across populations in the current study. That is that translocated populations tend to have lower gut microbiome alpha diversity compared to the source population, and that populations established with the fewest founders (Aride and Cousine) show reduced levels of inter‐individual gut microbiome variation.

It is also possible that variation in island area and overall population size could interact with environmental heterogeneity to influence gut microbiome differences across populations (Härer and Rennison 2023). For example, greater microbiome diversity might be expected in larger populations and those inhabiting larger islands since individuals that are spread over wider areas are likely to be exposed to greater habitat and microbial variation (Härer and Rennison 2023). However, although not all comparisons were significant, gut microbiome alpha diversity tended to be generally lower in translocated populations despite Aride, Denis and Frégate covering a greater absolute area than Cousin (0.68 km^2^, 1.42 km^2^, 2.19 km^2^ vs. 0.29 km^2^, respectively). Furthermore, Aride is inhabited by the largest population of warblers (*ca* 1850 individuals versus 210, 875, 561 and 320 individuals on Cousine, Denis, Frégate and Cousin respectively (Brown et al. 2023)) yet had one of the lowest levels of inter‐individual gut microbiome variability. Thus, although a greater number of populations would be needed to accurately assess the impact of island area and population size on gut microbiome variability, our results are not consistent with this being the main driver of differences across populations in this study.

### 4.3 Differential Abundance of Microbes

Many of the bacterial families that were differentially abundant between translocated population and Cousin can inhabit a wide range of niches outside of the gut. For example, most *Actinobacteria* are aerobic, spore‐forming microbes that are widely distributed across aquatic and soil environments (Barka et al. 2016). Similarly, proteobacterial families including *Acetobacteraceae*, *Beijerinckiaceae* and *Rhizobiaceae* are commonly found in association with soil, water and plants (Guzman and Vilcinskas 2022; Haque et al. 2020; Poole et al. 2018), and members of the *Burkholderiaceae* are known to be insect symbionts (Kaltenpoth and Flórez 2020). Thus, many of these bacteria could be transient colonisers of the gut, acquired from the external habitat or via the host's diet. Changes to their abundance across populations could therefore reflect variable uptake by warblers from different microbial pools across different islands. The wide niche breadth of these bacterial taxa may also explain why some families, such as the *Rhizobiaceae*, *Micrococcaceae* and *Microbacteriaceae*, were found ubiquitously in the core gut microbiome of all populations.

However, aside from aerobic, putatively ‘environmental’ microbes, several other families were lost from the core microbiome of translocated populations that are considered key components of the gut microbiome in other animals. This was particularly the case on Aride, whereby the families *Ruminococcaceae*, *Lachnospiraceae*, *Christensenellaceae*, *Akkermansiaceae* and *Tannerellaceae* (all core members of the Cousin Island gut microbiome) were all present in less than 35% of individuals and, thus, were not classified as core microbes. *Ruminococcaceae* and *Lachnospiraceae* are the most abundant and active members of the *Firmicutes* in the mammalian gut (Peris‐Bondia et al. 2011) and play an important role in the production of short chain fatty acids (SCFAs) like butyrate; such molecules contribute to gut epithelial tissue maintenance and are beneficial to host health (Fusco et al. 2023). Similarly, *Christensenallaceae* is one of the most highly heritable members of the human gut microbiome and the abundance of this family, as well as *Akkermansiaceae*, has been related to various aspects of metabolic health in mammals (Karcher et al. 2021; Waters and Ley 2019). It is unclear whether these bacterial families were replaced by other taxa capable of performing a similar function on Aride, Denis or Frégate. Two bacterial families in the phylum Firmicutes (*Leuconostocaceae* and *Lactobacillaceae*) formed novel components of the core microbiome on these islands. However, although these families are common in the intestinal tract and produce the SCFA lactic acid, they are also often found to be associated with insects and plants and so may be environmental transients (Endo et al. 2015; Walter and O'Toole 2023).

On Cousine, the *Bacteroidaceae* and *Rickenellaceae* formed part of the core microbiome but these families were not present in the Cousin Island core. Both families degrade complex sugars and proteins in the intestinal tract and are important producers of SCFAs (Rajilić‐Stojanović and De Vos 2014). Thus, there may be some degree of flexibility in the core microbiome across populations if distinct microbial groups perform similar tasks within the gut ecosystem. This flexibility was also evidenced by an indicator analysis which showed that unique ASVs within the same genus/family were indicative of different populations. However, without a detailed assessment of gut microbiome function across islands it is only possible to speculate on the extent to which functions are interchangeable across microbial groups. In future, high resolution functional omics approaches (e.g., metagenomics), would be needed to assess whether gut microbiome compositional differences across populations translate into differences in function and whether this could have consequences for the host (Worsley, Videvall, et al. 2024). Although translocated populations appear to be doing well on the different islands (i.e., at carrying capacity or still growing (Brown et al. 2023)) reductions in gut microbiome alpha diversity and functional potential could impact upon the resilience of these populations to future change. Longitudinal studies that include measures of gut microbiome variation and host fitness on these islands would be needed to assess if this is the case. Currently translocated populations are not subject to yearly monitoring and so this was beyond the scope of this study.

### 4.4 Study Limitations and Future Work

Although our results shed light on the impact of conservation translocations on the vertebrate gut microbiome there are some limitations. Aside from the lack of functional and long‐term fitness data already mentioned, the moderate sample sizes across populations may have hampered our ability to accurately capture the core microbiome on each island. Furthermore, although we identify differences in the gut microbiome of the translocated populations, it is impossible to know when these changes occurred; were changes the immediate result of founder effects or have gut microbiomes gradually diverged due to downstream host genetic effects and/or as individuals have been exposed to differing environments? Collecting samples directly before and periodically after translocations would be an interesting way to quantify the plasticity of the microbiome, and its susceptibility to founder effects (Grieneisen et al. 2023). Unfortunately, this was not feasible in the Seychelles warbler system as translocations occurred many years before gut microbiome samples were collected. That said, patterns of reduced gut microbiome diversity and compositional variability in translocated populations (particularly those with the fewest founders) are broadly consistent with an influence of host/microbiome founder effects.

## 5 Conclusion

In conclusion, our study identified a reduction in gut microbiome diversity, as well as differences in gut microbiome composition and variability between translocated and source populations of the Seychelles warbler. Members of the core microbiome also differed across populations and important gut microbes were less prevalent in some translocated populations. Population bottlenecks, due to the small number of founder individuals used, may drive these patterns both indirectly via an impact on host genetic variation or directly, by influencing the pool of microbes transferred from the source population. Further work is needed to understand the impact of these differences on gut microbiome function. Longitudinal data from translocated populations is also needed to better understand the plasticity of the gut microbiome and the impact of microbiome diversity loss on the resilience of host–microbe interactions to future ecological change.

## Author Contributions

**Sarah F. Worsley:** conceptualization (equal), data curation (lead), formal analysis (lead), funding acquisition (equal), investigation (lead), methodology (lead), visualization (lead), writing – original draft (lead), writing – review and editing (equal). **Zoe Crighton:** formal analysis (supporting), writing – review and editing (equal). **Chuen Zhang Lee:** writing – review and editing (equal). **Terry Burke:** project administration (equal), writing – review and editing (equal). **Jan Komdeur:** project administration (equal), writing – review and editing (equal). **Hannah L. Dugdale:** funding acquisition (equal), project administration (equal), writing – review and editing (equal). **David S. Richardson:** conceptualization (equal), funding acquisition (equal), project administration (equal), writing – review and editing (equal).

## Funding

This work was supported by the Natural Environment Research Council, NE/S010939/1. Biotechnology and Biological Sciences Research Council, BB/T008717/1. Leverhulme Trust, ECF‐2023‐433.

## Ethics Statement

Fieldwork was carried out in accordance with local ethical regulation and agreements (UEA ethics approval ID ETH2223‐0665). The Seychelles Department of Environment and the Seychelles Bureau of Standards approved the fieldwork (permit number A0157).

## Conflicts of Interest

The authors declare no conflicts of interest.

## Supporting information


**Figure S1:** The relative abundance (%) of bacterial families in Seychelles warbler gut microbiome samples. Each vertical bar represents a separate faecal sample; bars are ordered according to the abundance of *Streptococcaceae*. Samples were collected from the source (CN = Cousin Island) and translocated populations (CE = Cousine, DS = Denis, FR = Frégate, AR = Aride), respectively. The year (2019, 2022 or 2023) and season (Major/Minor) in which samples were collected is also given. Families with a median relative abundance of < 0.1% are collapsed into the category ‘Other’.
**Figure S2:** Gut microbiome alpha diversity of Seychelles warblers sampled from translocated populations (CE = Cousine, DS = Denis, FR = Frégate, AR = Aride) and Cousin Island (CN, the source population). The year (2019, 2022 or 2023) and season (Major/Minor) in which samples were collected is also given. Sample sizes are given above each violin. Significant pairwise comparisons (*p*_*adj*_ < 0.05) in pairwise post hoc comparisons are indicated by *.
**Figure S3:** The distribution of structural zeros across populations of the Seychelles warbler. Structural zeros are defined as bacterial families that are completely absent in at least one population and were identified using ANCOM‐BC. Blue cells show families that are present in a population whilst white cells show families that are absent. The total number of structural zeros is indicated for each population. Labels represent the phylum, family and a unique number (1–112) for each taxon. CN19 & CN22 = Cousin sampled in 2019 and 2022, respectively; CE = Cousine; AR = Aride; DS = Denis; FR = Frégate.
**Table S1:** Results of models comparing gut microbiome alpha diversity across source and translocated populations of the Seychelles warbler. A total of *n* = 51 and *n* = 80 samples were analysed from source and translocated populations, respectively. Linear models with a gaussian distribution were used to model Shannon and Faith's phylogenetic diversity metrics (test statistic ‘*t*’), whereas a generalised linear model with a negative binomial distribution was used for observed ASV richness (test statistic ‘*z*’). Reference categories for categorical variables are as follows: source (island type), female (sex).
**Table S2:** Models comparing Seychelles warbler gut microbiome alpha diversity across translocated populations (Cousine, Denis, Frégate, Aride) and their comparable sampling season on Cousin Island (the minor season of 2019 or major season of 2022, respectively). Linear models with a gaussian distribution were used to model Shannon and Faith's phylogenetic diversity metrics (test statistic *F*), whereas a generalised linear model with a negative binomial distribution was used for Observed ASV richness (test statistic *χ* ^2^). *N* = 20 samples from Cousine, Aride, Denis, Frégate and Cousin 2019, and *N* = 31 samples from Cousin 2022 were included in the analysis, respectively.
**Table S3:** The results of post hoc pairwise comparisons of gut microbiome alpha diversity for Seychelles warblers sampled from translocated populations (CE = Cousine, DS = Denis, FR = Frégate, AR = Aride) and Cousin Island (CN, the source population). Results are *p*‐values adjusted for multiple testing. Samples were collected in the minor season of 2019 (19 Minor) from CE and CN, the major seasons of 2022 (22 Major) from CN, DS and FR, and the major season of 2023 (23 Major) on AR, respectively. Separate models were run for Shannon diversity, observed ASV richness, and Faith's phylogenetic diversity (FPD), respectively (see Table S2). Significant comparisons (*p*_*adj*_ < 0.05) are highlighted in bold and underlined.
**Table S4:** Results of pairwise PERMANOVA analyses of gut microbiome composition between separate Seychelles warbler populations. CN = Cousin, CE = Cousine, DS = Denis, FR = Frégate, AR = Aride. The CN 19 and CE samples were collected in the minor season of 2019; CN 22, DS and FR samples were collected in the major season of 2022; AR samples were collected in the major season of 2023. The *p*‐values (top row) and *R* ^2^ values (bottom row) for the population model term are presented in the table (sex, time of day and storage time were also controlled for in analyses). Significant *p*‐values (*p*_*adj*_ < 0.05) are in bold and underlined (i.e., all *p*‐values are significant).
**Table S5:** Results of a permutational Betadisper test assessing differences in inter‐individual gut microbiome variance across populations. Numbers are adjusted *p*‐values derived from pair‐wise tests (significant tests are presented in bold and underlined). A significant *p*‐value indicates differences in inter‐individual compositional variance between population pairs. CN19 and CN22 = Cousin Island samples collected in 2019 and 2022, respectively; CE = Cousine; DS = Denis; FR = Frégate; AR = Aride.
**Table S6:** Differentially abundant bacterial families across source and translocated populations of the Seychelles warbler. Tests were carried out using ANCOM‐BC. A positive or negative log fold change (lfc) indicates that a family is more or less abundant in the first population listed in each comparison, respectively; the population with a greater abundance of each family is also shown in the final column for clarity. Only those with significant effect sizes (*p*_*adj*_ < 0.05) are shown. CN19 and CN22 = Cousin Island samples collected in 2019 and 2022, respectively; CE = Cousine; DS = Denis; FR = Frégate; AR = Aride.
**Table S7:** Results of an indicator species analysis across source and translocated populations of the Seychelles warbler. An indicator score (indval) of one indicates that an ASV is equally abundant in all samples from one population and effectively absent in other populations, whilst a score of zero would suggest approximately even abundance across samples from all populations. Amplicon sequencing variants (ASVs) with an indval of > 0.4 and *p* < 0.05 were considered to be indicative of a population/sampling period and are presented here along with their taxonomic identity to genus level. CN19 and CN22 = Cousin Island samples collected in 2019 and 2022, respectively; CE = Cousine; DS = Denis; FR = Frégate; AR = Aride.

## Data Availability

All sequencing reads have been uploaded to the European Nucleotide Archive (ENA) under the following accession numbers: PRJEB47095 (Cousin Island samples collected in 2019); PRJEB67634 (Cousin Island samples collected in 2022); and PRJEB81863 (all samples from translocated populations). The scripts and metadata to reproduce all analyses and figures can be accessed via the GitHub repository, https://github.com/Seychelle‐Warbler‐Project, and are also archived in the Dryad repository https://doi.org/10.5061/dryad.4mw6m90rf (Worsley, Crighton, et al. 2024).
