# Supplementary material for: Gut Microbiome Communities Vary Across Translocated Populations of the Seychelles Warbler
Source: Ecol Evol. 2026 May 29;16(6):e73750. doi: 10.1002/ece3.73750 (PMC13240254; doi:10.1002/ece3.73750)
Supplement: Supplementary file 1 — Figure S1: The relative abundance (%) of bacterial families in Seychelles warbler gut microbiome samples. Each vertical bar represents a separate faecal sample; bars are ordered according to the abundance of Streptococcaceae. Samples were collected from the source (CN = Cousin Island) and translocated populations (CE = Cousine, DS = Denis, FR = Frégate, AR = Aride), respectively. The year (2019, 2022 or 2023) and season (Major/Minor) in which samples were collected is also given. Families with a median relative abundance of < 0.1% are collapsed into the category ‘Other’. Figure S2: Gut microbiome alpha diversity of Seychelles warblers sampled from translocated populations (CE = Cousine, DS = Denis, FR = Frégate, AR = Aride) and Cousin Island (CN, the source population). The year (2019, 2022 or 2023) and season (Major/Minor) in which samples were collected is also given. Sample sizes are given above each violin. Significant pairwise comparisons (p adj < 0.05) in pairwise post hoc comparisons are indicated by *. Figure S3: The distribution of structural zeros across populations of the Seychelles warbler. Structural zeros are defined as bacterial families that are completely absent in at least one population and were identified using ANCOM‐BC. Blue cells show families that are present in a population whilst white cells show families that are absent. The total number of structural zeros is indicated for each population. Labels represent the phylum, family and a unique number (1–112) for each taxon. CN19 & CN22 = Cousin sampled in 2019 and 2022, respectively; CE = Cousine; AR = Aride; DS = Denis; FR = Frégate. Table S1: Results of models comparing gut microbiome alpha diversity across source and translocated populations of the Seychelles warbler. A total of n = 51 and n = 80 samples were analysed from source and translocated populations, respectively. Linear models with a gaussian distribution were used to model Shannon and Faith's phylogenetic diversity metrics (test st [file ECE3-16-e73750-s001.docx]

**Supplementary Materials**

**Gut microbiome communities vary across translocated populations of the Seychelles warbler**

Sarah F. Worsley^1*^, Zoe Crighton^1^, Chuen Zhang Lee^1^, Terry Burke^2^, Jan Komdeur^3^, Hannah L. Dugdale^3,4^ & David S. Richardson^1,5*^

^1^ School of Biological Sciences, University of East Anglia, Norwich Research Park, Norfolk, NR4 7TJ, UK

^2^ Ecology and Evolutionary Biology, School of Biosciences, University of Sheffield, Sheffield, S10 2TN, UK

^3^ Groningen Institute for Evolutionary Life Sciences (GELIFES), University of Groningen, P.O. Box 11103, 9700 CC, Groningen, The Netherlands

^4^ Faculty of Biological Sciences, School of Biology, University of Leeds, Leeds LS2 9JT, UK

^5^ Nature Seychelles, Roche Caiman, Mahé, Republic of Seychelles

*Correspondence: s.worsley@uea.ac.uk, david.richardson@uea.ac.uk

**Table S1.** Results of models comparing gut microbiome alpha diversity across source and translocated populations of the Seychelles warbler. A total of n = 51 and n = 80 samples were analysed from source and translocated populations, respectively. Linear models with a gaussian distribution were used to model Shannon and Faith’s phylogenetic diversity metrics (test statistic “*t*”), whereas a generalised linear model with a negative binomial distribution was used for observed ASV richness (test statistic “*z*”). Reference categories for categorical variables are as follows: source (island type), female (sex).

| **Predictor** | **Estimate** | **SE** | ***t/z*** | ***P*** |
| --- | --- | --- | --- | --- |
| 1. **Shannon diversity** | | | | |
| Intercept | 3.848 | 0.405 | 9.490 | <0.001 |
| **Island type** | **-0.596** | **0.252** | **-2.362** | **0.020** |
| Sex | 0.004 | 0.205 | -0.216 | 0.983 |
| Time of day | -0.001 | 0.001 | -1.734 | 0.085 |
| Storage time | -0.006 | 0.006 | -1.038 | 0.301 |
| 1. **Observed ASV richness** | | | | |
| Intercept | 5.363 | 0.185 | 28.972 | <0.001 |
| **Island type** | **-0.233** | **0.115** | **-2.025** | **0.043** |
| Sex | 0.045 | 0.094 | 0.481 | 0.630 |
| Time of day | 3.278 | <0.001 | 0.131 | 0.896 |
| Storage time | <-0.001 | 0.003 | -0.158 | 0.874 |
| 1. **Faith’s phylogenetic diversity** | | | | |
| Intercept | 17.508 | 2.243 | 7.804 | <0.001 |
| **Island type** | **-3.844** | **1.396** | **-2.755** | **0.007** |
| Sex | 0.646 | 1.134 | 0.569 | 0.570 |
| Time of day | <-0.001 | 0.003 | -0.019 | 0.984 |
| Storage time | 0.003 | 0.031 | 0.098 | 0.922 |

**Table S2.** Models comparing Seychelles warbler gut microbiome alpha diversity across translocated populations (Cousine, Denis, Frégate, Aride) and their comparable sampling season on Cousin Island (the minor season of 2019 or major season of 2022, respectively). Linear models with a gaussian distribution were used to model Shannon and Faith’s phylogenetic diversity metrics (test statistic *F*), whereas a generalised linear model with a negative binomial distribution was used for Observed ASV richness (test statistic *χ^2^*). *N* = 20 samples from Cousine, Aride, Denis, Frégate, and Cousin 2019, and N = 31 samples from Cousin 2022 were included in the analysis, respectively.

| **Predictor** | **df** | ***F or χ^2^*** | ***P*** |
| --- | --- | --- | --- |
| 1. **Shannon diversity** | | | |
| **Population** | **5** | **2.839** | **0.018** |
| Sex | 1 | 0.082 | 0.775 |
| Time of day | 1 | 2.263 | 0.135 |
| Storage time | 1 | 0.604 | 0.438 |
| 1. **Observed ASV richness** | | | |
| Population | 5 | 10.539 | 0.061 |
| Sex | 1 | 0.114 | 0.736 |
| Time of day | 1 | 0.003 | 0.954 |
| Storage time | 1 | 0.519 | 0.471 |
| 1. **Faith’s phylogenetic diversity** | | | |
| **Population** | **5** | **3.861** | **0.003** |
| Sex | 1 | 0.329 | 0.567 |
| Time of day | 1 | 0.089 | 0.766 |
| Storage time | 1 | 1.601 | 0.208 |

**Table S3.** The results of post-hoc pairwise comparisons of gut microbiome alpha diversity for Seychelles warblers sampled from translocated populations (CE = Cousine, DS = Denis, FR = Frégate, AR = Aride,) and Cousin Island (CN, the source population). Results are *P*-values adjusted for multiple testing. Samples were collected in the minor season of 2019 (19 Minor) from CE and CN, the major seasons of 2022 (22 Major) from CN, DS and FR, and the major season of 2023 (23 Major) on AR, respectively. Separate models were run for Shannon diversity, observed ASV richness, and Faith’s phylogenetic diversity (FPD), respectively (see Table S2). Significant comparisons (*P_adj_* < 0.05) are highlighted in bold and underlined.

| **Post-hoc comparison** | **Shannon** | **ASV richness** | **FPD** |
| --- | --- | --- | --- |
| CN 22 Major – CN 19 Major | 0.712 | 0.991 | 1.000 |
| CE 19 Minor – CN 19 Minor | 0.372 | 1.000 | 0.998 |
| DS 22 Major – CN 19 Minor | 0.997 | 0.999 | 1.000 |
| FR 22 Major – CN 19 Minor | 1.000 | 0.058 | **0.015** |
| AR 23 Major – CN 19 Minor | 0.999 | 0.945 | 0.965 |
| CE 19 Minor – CN 22 Major | **0.009** | 0.999 | 0.994 |
| DS 22 Major – CN 22 Major | 0.599 | 1.000 | 0.999 |
| FR 22 Major – CN 22 Major | 0.665 | 0.170 | **0.010** |
| AR 23 Major – CN 22 Major | 0.752 | 0.986 | 0.941 |
| DS 22 Major – CE 19 Minor | 0.434 | 0.999 | 0.909 |
| FR 22 Major – CE 19 Minor | 0.479 | 0.352 | 0.052 |
| AR 23 Major – CE 19 Minor | 0.532 | 0.820 | 0.521 |
| FR 22 Major – DS 22 Major | 0.999 | 0.660 | 0.394 |
| AR 23 Major – DS 22 Major | 1.000 | 0.937 | 0.953 |
| AR 23 Major – FR 22 Major | 1.000 | 0.974 | 0.850 |

**Table S4.** Results of pairwise PERMANOVA analyses of gut microbiome composition between separate Seychelles warbler populations. CN= Cousin, CE Cousine, DS = Denis, FR = Frégate, AR = Aride. The CN 19 and CE samples were collected in the minor season of 2019; CN 22, DS and FR samples were collected in the major season of 2022; AR samples were collected in the major season of 2023. The *P*-values (top row) and R^2^ values (bottom row) for the population model term are presented in the table (sex, time of day, and storage time were also controlled for in analyses). Significant *P*-values (*P_adj_* < 0.05) are in bold and underlined (i.e all *P*-values are significant).

|  | **CN 19** | **CN 22** | **CE** | **DS** | **FR** | **AR** |
| --- | --- | --- | --- | --- | --- | --- |
| **CN 22** | **0.006** |  |  |  |  |  |
|  | 0.035 |  |  |  |  |  |
| **CE** | **0.001** | **0.001** |  |  |  |  |
|  | 0.056 | 0.065 |  |  |  |  |
| **DS** | **0.001** | **0.001** | **0.001** |  |  |  |
|  | 0.097 | 0.067 | 0.148 |  |  |  |
| **FR** | **0.001** | **0.001** | **0.001** | **0.001** |  |  |
|  | 0.068 | 0.050 | 0.096 | 0.064 |  |  |
| **AR** | **0.001** | **0.001** | **0.001** | **0.001** | **0.001** |  |
|  | 0.092 | 0.050 | 0.153 | 0.079 | 0.084 |  |

**Table S5.** Results of a permutational Betadisper test assessing differences in inter-individual gut microbiome variance across populations. Numbers are adjusted *P*-values derived from pair-wise tests (significant tests are presented in bold and underlined). A significant *P*-value indicates differences in inter-individual compositional variance between population pairs. CN19 and CN22= Cousin Island samples collected in 2019 and 2022, respectively; CE= Cousine; DS = Denis; FR= Frégate; AR= Aride.

|  | **CN 19** | **CN 22** | **CE** | **DS** | **FR** | **AR** |
| --- | --- | --- | --- | --- | --- | --- |
| **CN 22** | 0.589 |  |  |  |  |  |
| **CE** | **0.032** | **0.003** |  |  |  |  |
| **DS** | 0.797 | 0.405 | **0.047** |  |  |  |
| **FR** | 0.371 | 0.116 | 0.171 | 0.507 |  |  |
| **AR** | 0.518 | **0.026** | 0.505 | 0.195 | 0.518 |  |

**Table S6.** Differentially abundant bacterial families across source and translocated populations of the Seychelles warbler. Tests were carried out using ANCOM-BC. A positive or negative log fold change change (lfc) indicates that a family is more or less abundant in the first population listed in each comparison, respectively; the population with a greater abundance of each family is also shown in the final column for clarity. Only those with significant effect sizes (*P_adj_* < 0.05) are shown. CN19 and CN22= Cousin Island samples collected in 2019 and 2022, respectively; CE= Cousine; DS = Denis; FR= Frégate; AR= Aride.

| **Family** | **Population comparison** | **lfc** | **se** | ***P_adj_*** | **Result** |
| --- | --- | --- | --- | --- | --- |
| JG30-KF-CM45 | DS v AR | -3.272 | 0.951 | 0.049 | >AR |
| Kineosporiaceae | DS v AR | -3.219 | 0.850 | 0.013 | >AR |
| Leuconostocaceae | CE v AR | -7.110 | 1.036 | <0.001 | >AR |
| Leuconostocaceae | CN19 v AR | -6.279 | 1.542 | 0.004 | >AR |
| Leuconostocaceae | CN22 v AR | -7.032 | 1.335 | <0.001 | >AR |
| Leuconostocaceae | DS v AR | -4.908 | 0.954 | <0.001 | >AR |
| Leuconostocaceae | FR v AR | -6.876 | 1.617 | 0.002 | >AR |
| Rhodobacteraceae | DS v AR | -3.317 | 0.937 | 0.034 | >AR |
| Akkermansiaceae | FR v CE | -5.831 | 1.641 | 0.032 | >CE |
| Bacteroidaceae | CE v AR | 4.088 | 1.071 | 0.011 | >CE |
| Dysgonomonadaceae | CE v AR | 3.762 | 0.966 | 0.008 | >CE |
| Dysgonomonadaceae | FR v CE | -5.070 | 1.445 | 0.037 | >CE |
| Rhodospirillaceae | CE v AR | 5.218 | 1.238 | 0.002 | >CE |
| Rikenellaceae | CE v AR | 3.683 | 0.921 | 0.005 | >CE |
| Rikenellaceae | FR v CE | -4.847 | 1.396 | 0.043 | >CE |
| Ruminococcaceae | CE v AR | 4.423 | 1.195 | 0.018 | >CE |
| Tannerellaceae | CE v AR | 4.364 | 1.134 | 0.010 | >CE |
| Tannerellaceae | DS v CE | -4.386 | 1.215 | 0.025 | >CE |
| Tannerellaceae | FR v CE | -5.831 | 1.631 | 0.029 | >CE |
| Desulfovibrionaceae | DS v AR | 4.172 | 1.078 | 0.009 | >DS |

**Table S7.** Results of an indicator species analysis across source and translocated populations of the Seychelles warbler. An indicator score (indval) of one indicates that an ASV is equally abundant in all samples from one population and effectively absent in other populations, whilst a score of zero would suggest approximately even abundance across samples from all populations. Amplicon sequencing variants (ASVs) with an indval of >0.4 and *P*<0.05 were considered to be indicative of a population/sampling period and are presented here along with their taxonomic identity to genus level. CN19 and CN22= Cousin Island samples collected in 2019 and 2022, respectively; CE= Cousine; DS = Denis; FR= Frégate; AR= Aride.

| **ASV ID** | **Population** | **Indval** | ***P*** | **Phylum** | **Family** | **Genus** |
| --- | --- | --- | --- | --- | --- | --- |
| 465c3a41559c565  5c0e1fa88362daf68 | CN 19 | 0.402 | 0.001 | Firmicutes | Lachnospiraceae |  |
| b3d2cc3b9f01848  30bec2ef73bcc6166 | CN 19 | 0.414 | 0.001 | Firmicutes | Streptococcaceae | Lactococcus |
| c95924232174923  7c9361696a94b439f | CN 22 | 0.453 | 0.002 | Actinobacteria | Kineosporiaceae | Kineococcus |
| d46e2205f0c6ecf67  b51f83d111c509c | CN 22 | 0.530 | 0.002 | Proteobacteria | Enterobacteriaceae | Escherichia-  Shigella |
| 078d853cea8dbb4e  06d303b1030ca618 | CE | 0.408 | 0.001 | Bacteroidetes | Tannerellaceae |  |
| e0b0346833a28530  10924b38ae0af05c | CE | 0.439 | 0.001 | Proteobacteria | Rhodospirillaceae |  |
| a6ba1b35cfea82e59  c5e16f4097bae33 | CE | 0.443 | 0.001 | Firmicutes | Lachnospiraceae | Lachnoclostridium |
| 9eedc8f1c0fe8101b  f0e935f85c9bd8b | CE | 0.458 | 0.001 | Proteobacteria | Enterobacteriaceae |  |
| 69eb94f0ce3e8fbdf  f13ba9a5fc91a76 | CE | 0.532 | 0.001 | Bacteroidetes | Rikenellaceae |  |
| b693ce0be65f8fc12  fdb52d333ca4e84 | CE | 0.547 | 0.001 | Tenericutes |  |  |
| e166afb28dc7d02ad  14c2c5e8d0ec59a | CE | 0.550 | 0.001 | Proteobacteria | Enterobacteriaceae | Arsenophonus |
| 6ba43acb03ae795b3  e7e31f2be946006 | AR | 0.468 | 0.001 | Firmicutes | Leuconostocaceae | Leuconostoc |
| 3f73222e4a3ce752  1ef471683fe61079 | AR | 0.523 | 0.001 | Proteobacteria | Rhizobiaceae |  |
| 1f972b567fd709ea  28ec93a3de972d07 | AR | 0.743 | 0.001 | Firmicutes | Leuconostocaceae | Fructobacillus |
| 5143fce345d2e57b1  edd8828e557d088 | AR | 0.927 | 0.001 | Firmicutes | Leuconostocaceae | Fructobacillus |
| a9499372ae409848f  a2ed116e7289617 | DS | 0.400 | 0.001 | Proteobacteria | Desulfovibrionaceae | Desulfovibrio |
| 49ec7420d727adfe1  d82d23d6e7e1c97 | DS | 0.400 | 0.001 | Proteobacteria | Desulfovibrionaceae | Desulfovibrio |
| 4e9d340cd157a21ec  1867c1f23ffff33 | DS | 0.442 | 0.001 | Proteobacteria | Desulfobacteraceae | Desulfatiferula |
| 1aa398aee86017619  6ea988201ba2d38 | DS | 0.452 | 0.001 | Proteobacteria | Desulfovibrionaceae | Desulfovibrio |
| f47c3a3139f65e66  a59a54937d17cedb | DS | 0.491 | 0.001 | Firmicutes | Lachnospiraceae |  |
| 550d54a1386bfb7d  9df8076e69906b5a | DS | 0.600 | 0.001 | Firmicutes | Enterococcaceae | Catellicoccus |
| 6cba272853a7fc66  33d3cd4d8f292018 | DS | 0.665 | 0.001 | Actinobacteria | Micrococcaceae |  |
| bec9d3b0f492da35e  957bc19a7053e51 | FR | 0.450 | 0.001 | Actinobacteria |  |  |
| 31e139f7528db0f6  fa415a3084cb56c9 | FR | 0.478 | 0.001 | Actinobacteria | Tsukamurellaceae | Tsukamurella |

**
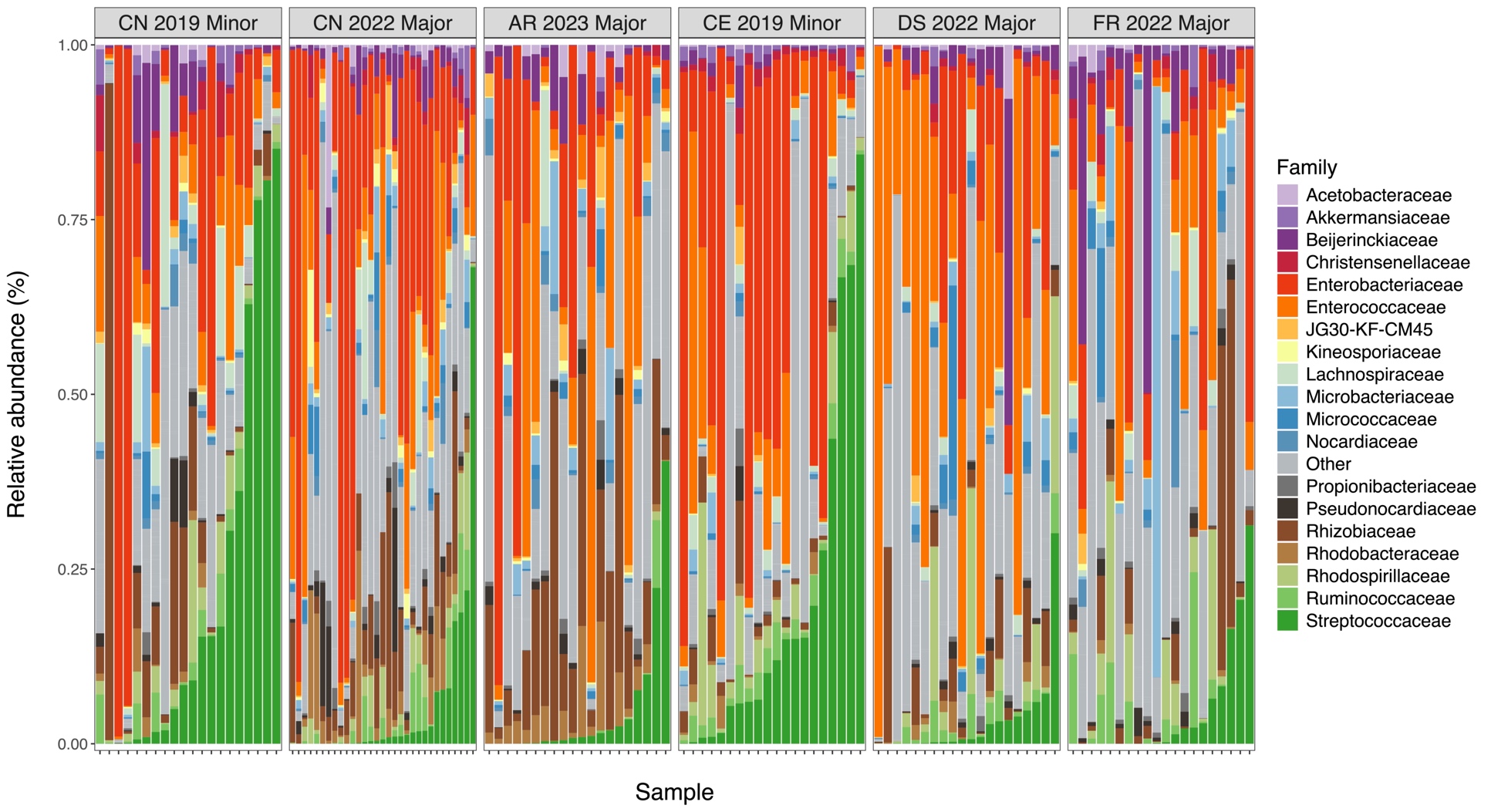
**

**Figure S1.** The relative abundance (%) of bacterial families in Seychelles warbler gut microbiome samples. Each vertical bar represents a separate faecal sample; bars are ordered according to the abundance of *Streptococcaceae*. Samples were collected from the source (CN= Cousin Island) and translocated populations (CE = Cousine, DS = Denis, FR = Frégate, AR = Aride), respectively. The year (2019, 2022, or 2023) and season (Major/Minor) in which samples were collected is also given. Families with a median relative abundance of less than 0.1% are collapsed into the category “Other”.

**Figure S2.** Gut microbiome alpha diversity of Seychelles warblers sampled from translocated populations (CE = Cousine, DS = Denis, FR = Frégate, AR = Aride,) and Cousin Island (CN, the source population). The year (2019, 2022, or 2023) and season (Major/Minor) in which samples were collected is also given. Sample sizes are given above each violin. Significant pairwise comparisons (*P_adj_* < 0.05) in pairwise post-hoc comparisons are indicated by *.

**
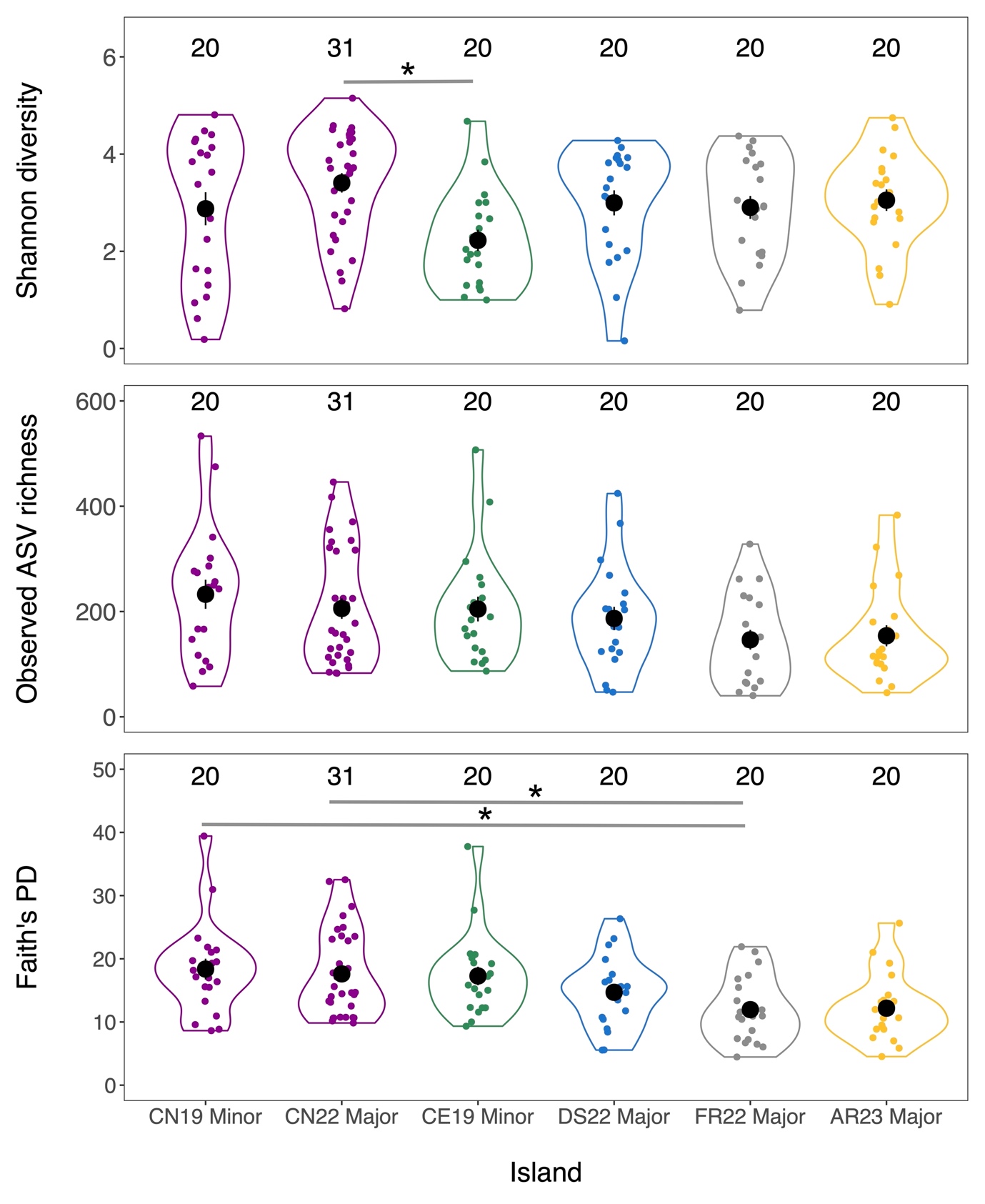
**

**Figure S3.** The distribution of structural zeros across populations of the Seychelles warbler. Structural zeros are defined as bacterial families that are completely absent in at least one population and were identified using ANCOM-BC. Blue cells show families that are present in a population whilst white cells show families that are absent. The total number of structural zeros is indicated for each population. Labels represent the phylum, family and a unique number (1-112) for each taxon. CN19 & CN22 = Cousin sampled in 2019 and 2022, respectively; CE = Cousine; AR= Aride; DS= Denis; FR = Frégate.
